# Tailoring the Band Structure of Twisted Double Bilayer Graphene with Pressure

**DOI:** 10.1021/acs.nanolett.1c03066

**Published:** 2021-10-18

**Authors:** Bálint Szentpéteri, Peter Rickhaus, Folkert K. de Vries, Albin Márffy, Bálint Fülöp, Endre Tóvári, Kenji Watanabe, Takashi Taniguchi, Andor Kormányos, Szabolcs Csonka, Péter Makk

**Affiliations:** † Department of Physics, Budapest University of Technology and Economics and Nanoelectronics Momentum Research Group of the Hungarian Academy of Sciences, Budafoki ut 8, 1111 Budapest, Hungary; △ Department of Physics, Budapest University of Technology and Economics and Correlated van der Waals Structures Momentum Research Group of the Hungarian Academy of Sciences, Budafoki ut 8, 1111 Budapest, Hungary; ‡ Solid State Physics Laboratory, ETH Zürich, CH-8093 Zürich, Switzerland; § Research Center for Functional Materials, National Institute for Materials Science, 1-1 Namiki, Tsukuba 305-0044, Japan; ∥ International Center for Materials Nanoarchitectonics, National Institute for Materials Science, 1-1 Namiki, Tsukuba 305-0044, Japan; ⊥ Department of Physics of Complex Systems, Eötvös Loránd University, Pázmány P. s. 1/A, 1117 Budapest, Hungary

**Keywords:** twisted double bilayer graphene, superlattice, pressure, band structure, transport measurements, continuum modeling

## Abstract

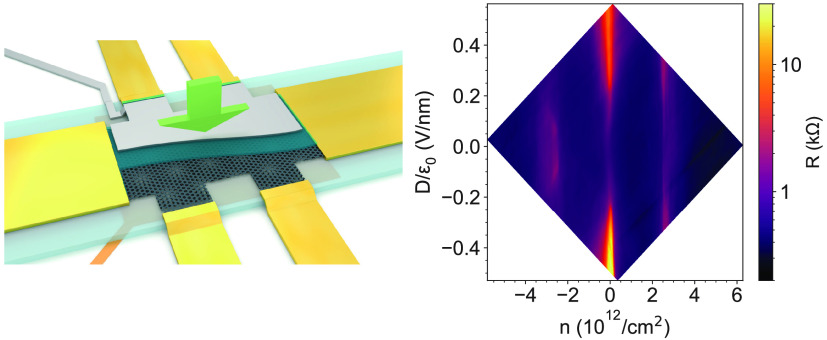

Twisted two-dimensional structures open new possibilities in band structure engineering. At magic twist angles, flat bands emerge, which gave a new drive to the field of strongly correlated physics. In twisted double bilayer graphene dual gating allows changing of the Fermi level and hence the electron density and also allows tuning of the interlayer potential, giving further control over band gaps. Here, we demonstrate that by application of hydrostatic pressure, an additional control of the band structure becomes possible due to the change of tunnel couplings between the layers. We find that the flat bands and the gaps separating them can be drastically changed by pressures up to 2 GPa, in good agreement with our theoretical simulations. Furthermore, our measurements suggest that in finite magnetic field due to pressure a topologically nontrivial band gap opens at the charge neutrality point at zero displacement field.

## Introduction

Twisted van der Waals heterostructures recently opened a new platform to explore correlated electronics phases. These structures consist of two or more layers of 2D materials, e.g., graphene, placed on top of each other, with a well-defined rotation angle between their crystallographic axes. The rotation of the two layers leads to the formation of a moiré superlattice. For small angles, the hybridization between the layers becomes significant, resulting in a reconstruction of the band structure, and for well-defined special, so-called ”magic” angles, the bands flatten out.^1,2^ Due to their narrow bandwidth, the electron–electron interaction can become comparable to the kinetic energy and novel, correlated phases form.^3−13^ The ability to control the size of the moiré unit cell size with the twist angle and implicitly the bandwidth of the low energy bands led to the discovery of various correlated phases^3,7−11^ such as correlated insulator states,^3−6,8^ ferromagnetic phase with a signature of the quantum anomalous Hall effect,^7−9,13^ and unconventional superconducting phases resembling high-temperature superconductors^4−6,12^ in twisted bilayer graphene (TBG). Moreover, the correlation effects are often accompanied by nontrivial topology of the bands.^13−25^

**Figure 1:**
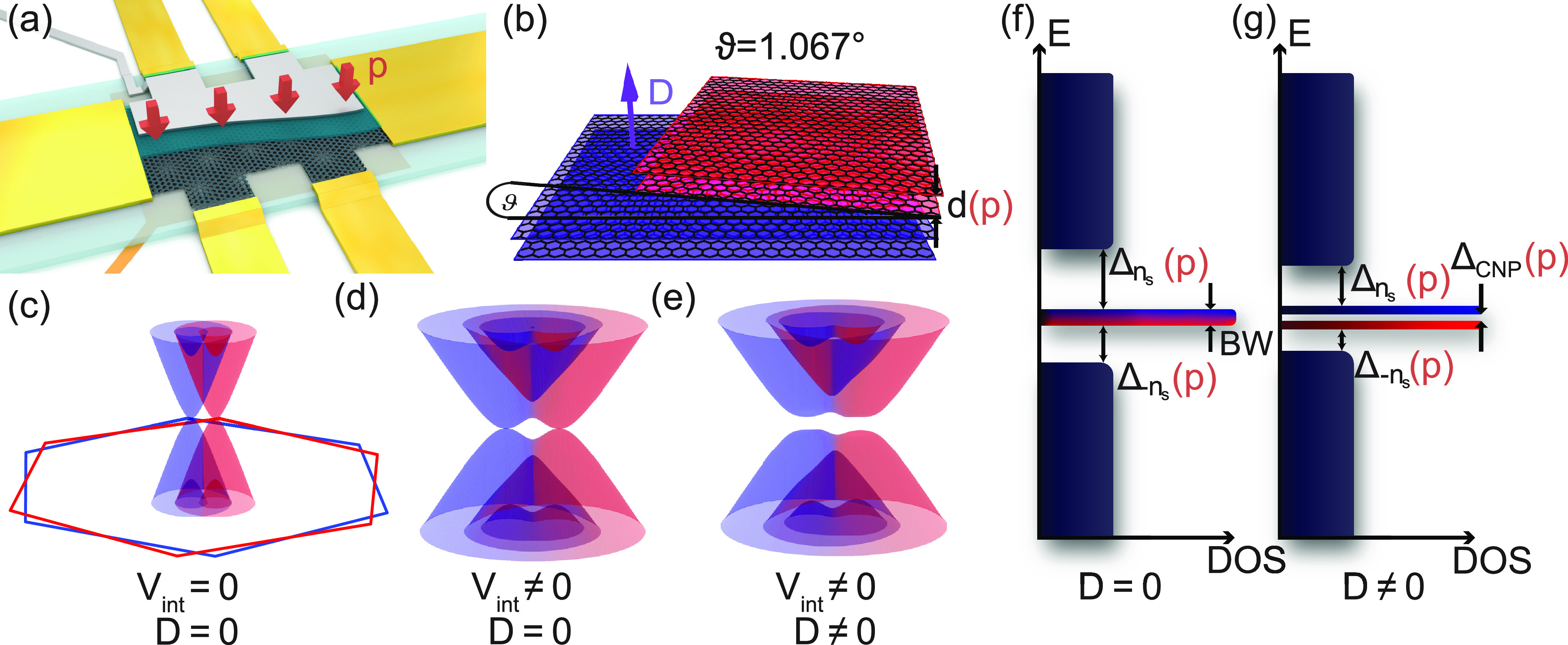
Properties of the twisted double bilayer graphene device. (a) Schematic of the TDBG (black lattice) with a bottom graphite gate (orange) isolated by a hBN layer (light blue) and a top metallic gate (gray) isolated by a hBN layer and an AlO_*x*_ layer (blue). The sample is contacted by edge contacts (yellow). The red arrows represent the pressure *p*, which modifies the distance between the layers. (b) Illustration of the twisted double bilayer structure. The purple arrow shows the direction of the transverse displacement field (*D*), ϑ is the twist angle, and *d* is the distance between the graphene layers which is tuned with the pressure. (c–e) Illustration of the band structure of TDBG in a corner of the Brillouin zone: (c) no coupling between the top and bottom BLGs which are depicted with red and blue colors, respectively; (d) a small coupling is introduced between the two BLGs which hybridizes the bands and leads to avoided crossings; (e) an external electric field can open a band gap at the Dirac points. (f, g) Schematic pictures of the DOS in the surrounding of the flat band in magic-angle TDBG without and with an electric field, respectively. The external electric field splits the degenerate flat bands and opens a gap Δ_CNP_ at the charge neutrality point. Δ_*n*_s__ and Δ_–*n*_s__ are the band gaps separating the flat bands from the conduction and valence bands. These gaps are tunable with external pressure which is noted explicitly by the parentheses.

Twisted double bilayer graphene (TDBG), which consists of two Bernal stacked bilayer graphene (BLG) crystals with a rotation angle of ϑ between them (depicted in Figure 1b), offers a versatile platform where an external electric field can be used to control the band structure via tuning of the interlayer potential. Recent experimental^24,26−31^ and theoretical^19−23,32,33^ studies showed the presence of correlated insulator states and topologically nontrivial phases in TDBG.

Since the reconstruction of the band structure and the appearance of the correlated phases depend on the interactions between the layers, these are extremely sensitive to the interlayer distance. Therefore, the tuning of the interlayer distance in these structures is of central interest. This can be achieved by applying external pressure (*p*), e.g., by using a hydrostatic pressure cell. The power of this method was demonstrated in graphene/hBN superlattices as a change of the superlattice potential,^34^ in layered antiferromagnets as an antiferromagnetic to ferromagnetic transition,^35−37^ and in Gr/WSe_2_ heterostructures, as an increase of induced spin–orbit coupling.^38,39^ In TBG the pressure changes the width of the low-energy bands^40,41^ and it can drive the system through a superconducting phase transition.^5^ On the basis of theoretical calculations on TDBG, the pressure is expected to have similarly drastic effects on the electronic properties;^20,32^ thus it gives an ideal control knob for in situ band-structure and topology engineering of TDBG.

In order to understand the influence of pressure on correlated phases in TDBG, the first step is to study the pressure dependence of the main parameters of the band structure in the single-particle picture. Here, we report for the first time the tuning of the band structure of twisted double bilayer graphene (TDBG) close to the magic angle (1.05°) by the application of hydrostatic pressure. Using bias spectroscopy and thermal activation measurements, we demonstrate a strong modulation of the single-particle band gaps of the system, which can be fully closed for 2 GPa of pressure. These findings agree well with our band-structure calculations. Moreover, our measurements indicate that pressure can lead to a topologically nontrivial gap at finite magnetic fields at the charge neutrality point.

## Schematic Description of the TDBG

Our TDBG device is fabricated with the tear-and-stack and dry stacking methods^42−44^ with a twist angle of ϑ = 1.067° (determined from quantum oscillations in magnetoconductance shown in the Supporting Information) and is encapsulated in hexagonal boron nitride (hBN). It has a graphite bottom and a metallic top gate as shown schematically in Figure 1a. The details of fabrication and an optical image of the device can be found in Methods and the Supporting Information. This dual gated geometry allows us an independent control of the charge density (*n*) and the transverse electric displacement field (*D*) in the TDBG.

The rotation between the top and bottom BLG lattices, illustrated in Figure 1b, leads also to a rotation between their Brillouin zones (BZ). These, along with the simplified BLG spectrum of each lattice, are presented in Figure 1c with red and blue for the top and bottom bilayers, respectively. For small rotation angles, the spectra of the bottom and top bilayers overlap. The coupling *V*_int_ between the closest monolayers of the BLGs hybridizes the bands and leads to avoided crossings as shown in Figure 1d. Moreover, at magic twist angles the low-energy moiré bands become flat^1,45−47^ due to the strong interlayer coupling driven avoided crossings. This is illustrated with the density of states (DOS) in Figure 1f. The flat bands (red and blue) have a small bandwidth (BW) and are separated from the dispersive conduction bands by a gap Δ_*n*_s__ and from the valence bands by Δ_–*n*_s__. Here *n*_s_ is the carrier density required to fill a single moiré band, either red or blue, with 4-fold degeneracy corresponding to four holes or electrons per superlattice unit cell in real space due to the spin and valley degeneracy. The indices of the gaps Δ_±*n*_s__ signify that in order to fill either flat band and move the Fermi level into either gap, the carrier density must be ±*n*_s_.

In the TDBG the external perpendicular displacement field *D* is an additional control parameter to tune the band structure and can open a gap Δ_CNP_ at the charge neutrality point^13,19,20^ as depicted in Figure 1; e.g., the flat bands split into two 4-fold degenerate bands, and the gaps separating them from the dispersive bands Δ_*n*_s__ and Δ_–*n*_s__ are decreased.

In the following let us focus on how the pressure controls the BW, the single particle gaps, and Δ_CNP_ of TDBG.

## Results and Discussion

**Figure 2:**
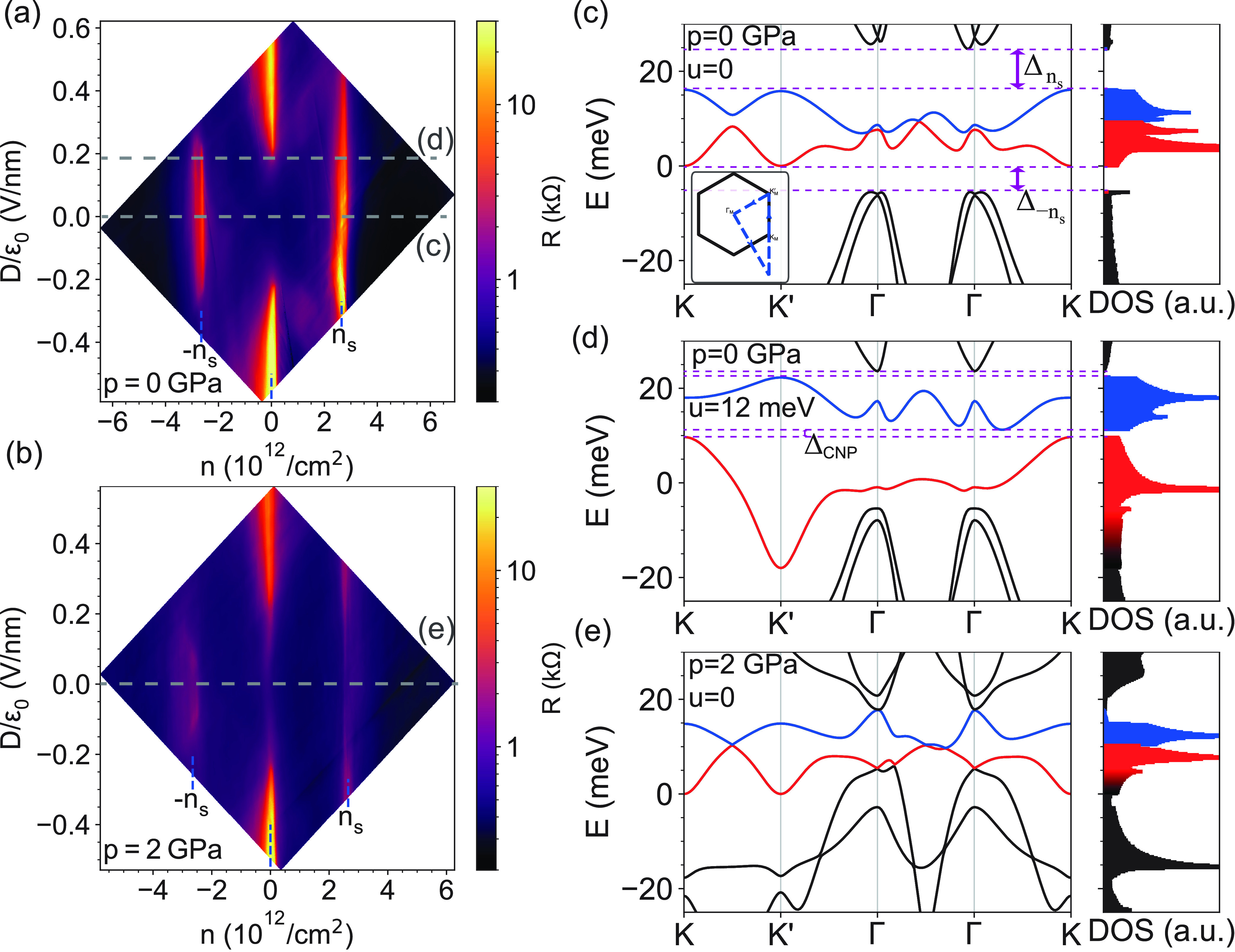
Device characterization and band structure calculation. (a, b) Four-probe resistance of the TDBG as a function of the charge density (*n*) and electric displacement field (*D*) measured (a) at ambient pressure *p* and (b) at *p* = 2 GPa. Besides the charge neutrality point, there are two other high resistance regions at ±*n*_s_, when the flat bands are completely filled. (c–e) Calculated band structure and DOS of the TDBG at ϑ = 1.067° twist angle, roughly corresponding to the dashed lines in (a) and (b). The flat bands are highlighted with red and blue colors. The spectra in (c) and (d) are calculated at ambient pressure for displacement fields *D* = 0 and *D*/ϵ_0_ ≈ 0.18 V/nm. A discussion on conversion of interlayer potential *u* to displacement field is given later and in the Supporting Information. The band structure in (e) is calculated for *D* = 0, *p* = 2 GPa. In the inset of (c) the moiré-Brillouin zone is shown.

Figure 2a shows a four-probe resistance (*R*_*xx*_) measurement as a function of top and bottom gate voltages, plotted as a function of electron density *n* and displacement field *D* at temperature *T* = 1.5 K. Lighter colored regions of higher resistance correspond to conditions when the Fermi energy is in a gap. If the flat bands are completely filled with electrons or holes at *n* = ±*n*_s_ the device shows single-particle gaps which are the most prominent at *D* = 0 and start to fade away for larger displacement fields. Moreover, at the charge neutrality point (CNP, *n* = 0), a gap opens by increasing |*D*| as demonstrated by the increase of the resistance with the increase of |*D*| in Figure 2a. This can be well explained by our band structure calculations done using a continuum model.

We calculated the band structure of the TDBG at ϑ = 1.067° using the noninteracting Bistritzer–Macdonald model^1^ using the parameters from refs (20) and (48). First, in Figure 2c we show the low-energy band structure for *D* = 0 and zero external pressure (*p* = 0), along a path in the moire-Brillouin zone indicated by the dashed line in the inset. The flat bands are depicted in red and blue colors. We denote the gaps separating the flat bands from the dispersive ones by Δ_±*n*_s__. Large resistance is expected when the Fermi energy is in the gapped regions of the band structure. A calculated spectrum for finite *D* and zero pressure is shown in Figure 2d. On one hand, *D* opens a band gap Δ_CNP_ at the CNP between the two flat bands. On the other hand, it decreases and eventually closes the gaps Δ_±*n*_s__. Overall, the band structure calculations are in agreement with the measurements presented in Figure 2a.

As a next step we applied hydrostatic pressure of 2 GPa on the sample (see Methods) and in a subsequent cooldown to 1.5 K, *R*_*xx*_ was remeasured as a function of the gate voltages. The result is shown in Figure 2b with the same color scale as in Figure 2a. The features are similar as at *p* = 0 except that the resistance values at *n* = ±*n*_s_ are significantly smaller than in Figure 2a. For example along *n* = −*n*_s_ the resistance decreased by 70% at *p* = 2 GPa. To understand the origin of this change, we calculated the evolution of the band structure using the pressure dependence of the interlayer coupling parameters given in ref (20). These parameters increased by ≈30% at *p* = 2 GPa from their value at *p* = 0 while the interlayer distance decreased by ≈5%. The spectrum at *p* = 2 GPa and zero displacement field is plotted in Figure 2e. As a result of pressure, the flat bands slightly narrow down and the dispersive bands shift down (up) in energy for the electron (hole) side and close the gaps at ±*n*_s_. (Later, we show the *D* dependence of the gaps at *p* = 2 GPa.) Moreover, in Figure 2a there is a sign of emerging correlated phases at half-filling, which disappears at *p* = 2 GPa (see Figure 2b.), similar to what was observed in magic-angle bilayer graphene.^5,40,49^

**Figure 3:**
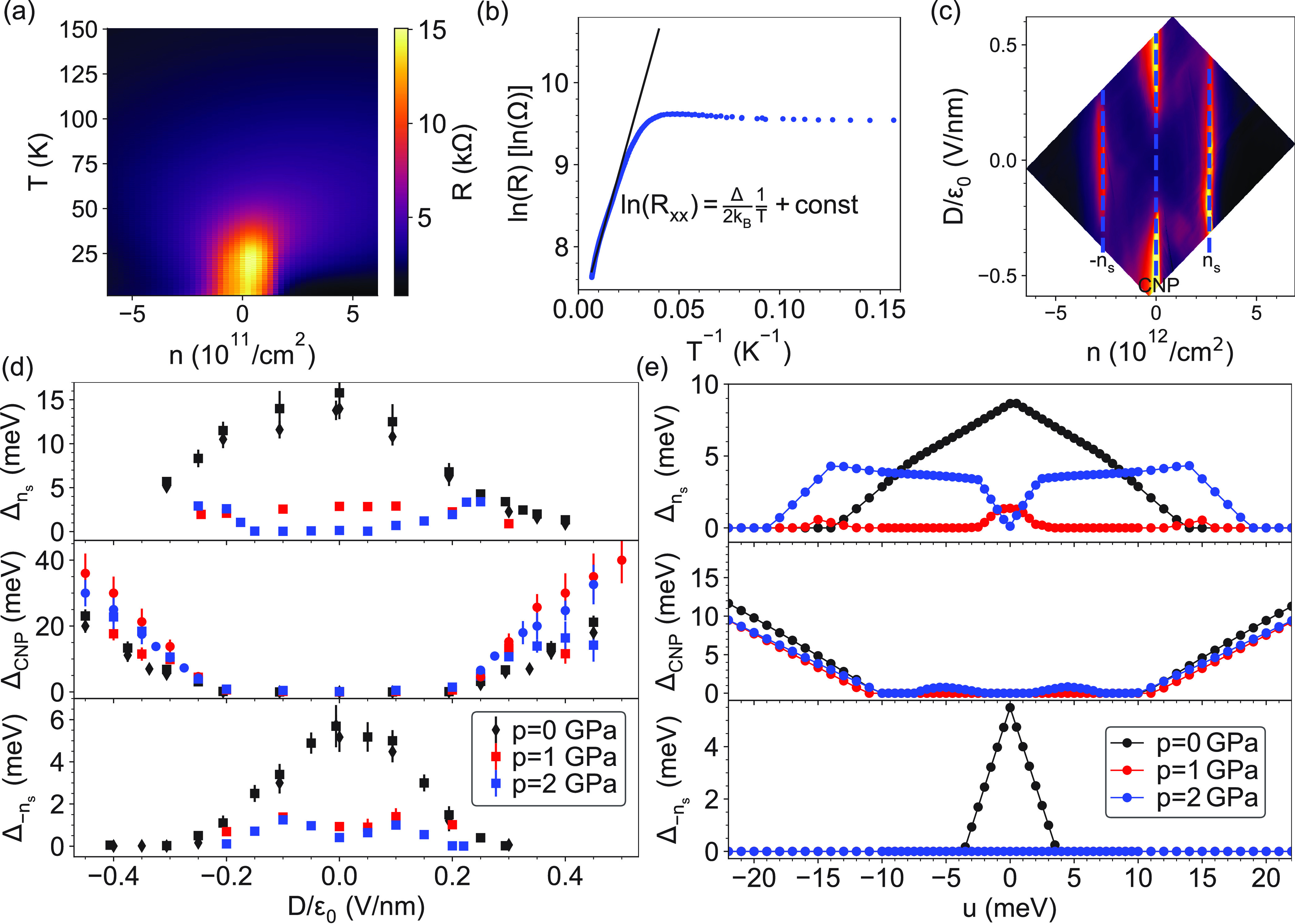
Thermal activation measurements of band gaps and comparison to the theoretical results. (a) Typical resistance map measured at *D*/ϵ_0_ = 0.375 V/nm and *p* = 0 near *n* = 0 as a function of the temperature *T* and charge density *n*. At every measured temperature the highest resistance value was taken. (b) *R* peaks extracted from (a) as a function of 1/*T*. The black line is the fit from which the gap values were obtained according to the Arrhenius equation. (c) The same *n*–*D* map of the resistance as Figure 1a showing the lines along which gap values were estimated for several *D* values in (d) using similar thermal activation measurements as presented in (b). (d) Measured gaps with respect to *D*. The different colors show the gaps at different pressures. The gaps obtained from different methods are shown with different markers: the squares and diamonds show gaps obtained from four-probe and two-probe thermal activation measurements, respectively, and the circles are gaps obtained from bias measurements. (e) Corresponding theoretically calculated single-particle gaps at three different pressures with respect to the on-site energy difference (*u*) of the layers.

To quantitatively verify the pressure dependence of the band structure and to extract the gap sizes, we performed thermal activation measurements. For this purpose, we measured the four-terminal resistance at a fixed *D* in a small range of *n* near the gapped regions as a function of the temperature (*T*). In Figure 3a we show a typical activation measurement, i.e., a resistance map as a function of *n* and *T*. The resistance decreases with increasing temperature due to thermal activation over the gap. To determine the gap size, we extract the resistance maximum for each temperature value, *R*_*xx*_(*T*) . Then the gap value was extracted from Arrhenius plot, where the logarithm of *R*_*xx*_(*T*) was plotted as a function of *T*^–1^ (see Figure 3b). The linear region close to zero in the *x*-axis is used to extract the gap energies by the Arrhenius equation (, where *k*_B_ is the Boltzmann constant). The slope of the linear fit (black line) provided the gap values. Error bars originate from the uncertainties in the fitting temperature range.

The gap energies were extracted in this way at several *D* points along the three dashed lines shown in Figure 3c, which are denoted as ±*n*_s_ and CNP. These data are shown in Figure 3d at three different pressures with three different colors. The middle panel presents the evolution of the gap with *D* at the CNP (Δ_CNP_). The system is not gapped at *D* = 0 and a gap opens at *D*/ϵ_0_ ≈ 0.2 V/nm where ϵ_0_ is the vacuum permittivity. The top panel shows the *D* dependence of the moiré gap at the electron side. A nonzero gap is present at *D* = 0, which decreases with |*D*| until it closes at |*D*|/ϵ_0_ ≈ 0.4 V/nm. The bottom panel summarizes the behavior of the gap at −*n*_s_ (Δ_–*n*_s__). It behaves similarly to Δ_*n*_s__ as it is finite at zero displacement field and decreases with |*D*|, but it closes at a smaller displacement field (|*D*|/ϵ_0_ ≈ 0.3 V/nm). The results at ambient pressure are in accordance with the previous findings.^20,21,26,28,31^ The measurement results obtained at pressures of *p* = 1 GPa and *p* = 2 GPa are shown in the same plots with red and blue colors, respectively. At finite pressure Δ_CNP_ behaves similar to *p* = 0: the gap opens around the same displacement field (middle panel). However, for Δ_±*n*_s__ the displacement field dependence strongly deviates from results at ambient pressure. At 1 GPa (red symbol) Δ_*n*_s__ is smaller than at *p* = 0 by a factor of 4 but follows the same tendency. On the other hand, at 2 GPa (blue symbol) Δ_*n*_s__ is closed at *D* = 0 and opens at finite *D*. The Δ_–*n*_s__ data at *p* = 1 GPa and *p* = 2 GPa are similar to each other: they exhibit a peak at finite |*D*| before decaying, but they are strongly reduced compared to the *p* = 0 values.

We note that the gap energies remained approximately the same at the same hydrostatic pressures during different cooldowns and pressurization cycles. We confirmed a part of the thermal activation measurements at 2 and 1 GPa using bias spectroscopy shown by circle symbols (for details see the Supporting Information). These results are generally consistent with the thermal activation data.

A good qualitative understanding of the pressure dependence of the measured gaps can be obtained by comparing them to our theoretical calculations shown in Figure 3e. In these calculations we neglected the electron–electron interactions and the quantum capacitance. The band gaps as a function of the interlayer potential difference (*u*), which is proportional to the displacement field, are plotted in Figure 3e. The gaps in the measurements show qualitatively the same dependence on the displacement field as our calculation, and the measured gap values are also comparable to the calculated ones. In our calculations, for Δ_*n*_s__, the gap values at ambient pressure and at *p* = 1 GPa decrease as a function of *u*, the latter being smaller than the former. Furthermore, the *p* = 1 GPa gap closes for smaller *u*. At *p* = 2 GPa the gap opens with *u* and closes at a higher interlayer potential difference. This tendency is similar to the experimental results. However, the theory suggests a faster opening of the gap at 2 GPa compared to the experiment. For the hole sides, at ambient pressure a finite gap is present in the calculations, which closes by increasing *u*. In contrast, the gap is absent both for *p* = 1 GPa and *p* = 2 GPa for all interlayer potential difference. These tendencies are qualitatively the same as observed in the experiments where only small gaps are seen at large pressure. Altogether, the measured spectrum is in good agreement with the theoretical expectations considering the simplicity of our model which is discussed later.

In order to compare the interlayer potential to the displacement fields applied in the experiment, we used the relation of , where *d* = 0.33 nm is the interlayer distance of bilayer graphene and ϵ = 6 ± 2 ^50,51^ is the relative dielectric constant of bilayer graphene. Using ϵ = 5 gives a relatively good agreement for the gap at the CNP and less good for the moiré gaps (see Supporting Information Figure S9). We emphasize that as a simplification we have used equal potential drop between all four layers in the calculations. However, the potential drop between the layers can be different originating from crystal fields^52^ and the different layer distances and dielectric constants. Moreover, we have neglected all quantum capacitance corrections and most importantly all correlation effects in the calculations (see Supporting Information).

**Figure 4:**
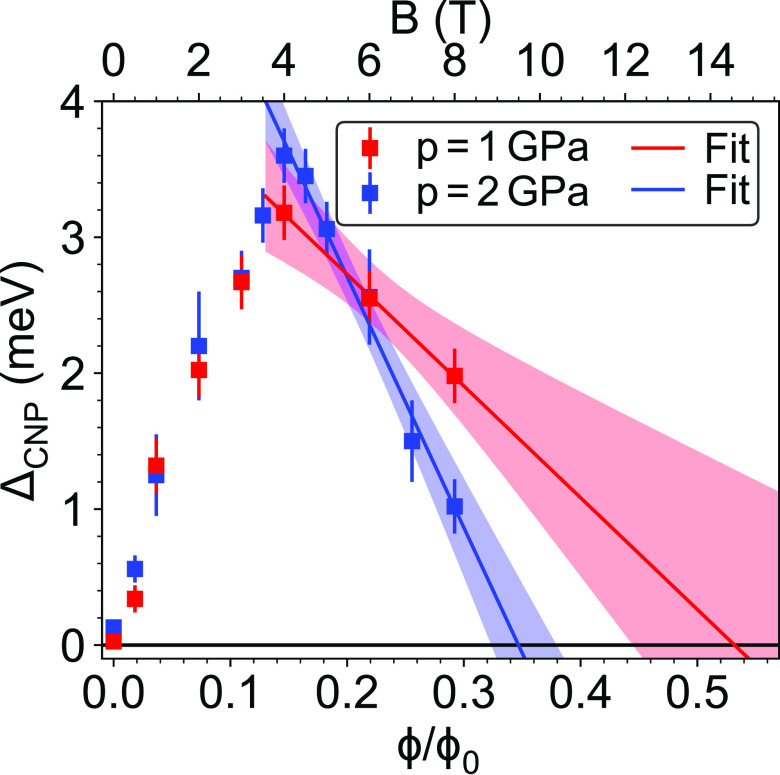
Charge neutrality gap (*n* = 0) at *D* = 0 as a function of the magnetic flux in the superlattice unit cell (Φ = *BA*_s_). The corresponding magnetic field *B* is given on the top axis. The gap starts to close above ∼4 T and closes completely approximately at Φ/Φ_0_ = 1/3 for 2 GPa. The blue and red lines are extrapolations for the gap at *p* = 2 GPa and *p* = 1 GPa, respectively. The shaded areas are the 95% confidence interval of the extrapolation.

Finally, we also find signatures of interesting topological effects at the CNP (*D* = 0) in an out-of-plane magnetic field. By increasing of the magnetic field *B*, a gap opens, and surprisingly above ∼4 T this gap starts to close. This is shown in Figure 4 for 1 and 2 GPa, respectively. This finding is similar to the recent results by Burg et al. in ref (24), where the authors argued that if the gap is nontrivial with a nonzero Chern number (*C*), then the gap should close at Φ/Φ_0_ = 1/|*C*|. Here Φ = *BA*_s_ is the magnetic flux penetrating the superlattice unit cell, *A*_s_ is the area of the superlattice unit cell which is given by eq S2 in the Supporting Information, and ϕ_0_ = *h*/*e* is the flux quantum with Planck’s constant *h* and the elementary charge *e*. The measurements of ref (24) were performed at ambient pressure using a sample of ϑ = 1.01° twist angle. Their results suggested the presence of a nontrivial gap with *C* = 2 at finite *D*, which agrees with the theory,^21−23,53^ and similar gap opening and closing were observed for *D* = 0. Our device shows a similar behavior, however, for *D* = 0 and finite pressure as shown in Figure 4. At 2 GPa the gap closes near Φ/Φ_0_ = 1/3, which could suggest a band gap with a Chern number of *C* = 3. Surprisingly at 1 GPa (red symbols and red line) the gap starts to close at the same magnetic field as for 2 GPa, but the extrapolation suggests that it would go to zero at much higher magnetic field than accessible in our setup (8 T). This suggests that the Chern number may depend on the pressure. Another, more likely possibility stems from the decrease of correlations effects which is well visible in Figure 2, by the disappearance of correlated features at half filling at finite pressures. This will lead to a smaller value of Φ/Φ_0_ where the gap closes.^24^ For a better understanding, further studies are required both theoretically and experimentally.

## Conclusion

We investigated a TDBG with ϑ = 1.067° under pressure at different temperatures and magnetic fields. We found that the band structure significantly changes with pressure: the single-particle moiré gaps present at zero displacement fields can be fully closed. We compared our findings with a single-particle continuum model and found a reasonable agreement with our experimental data. The changes achievable with the pressure are large and on the order of the bandwidth of the central flat band. The large tunability and the theoretical predictability suggest that the band structure of twisted structures can be precisely designed by taking into account the modified layer separation. The pressure combined with the electric field tunability allows extensive control of the band structure in situ. The exploration of the single-particle band structure is an important milestone toward tuning and understanding the emerging topological and correlated states of these systems. In addition we showed that in out-of-plane magnetic field the closing of the gap at the charge neutrality point strongly depends on pressure, suggesting pressure dependence of the Chern number.

## Methods

### Device Fabrication

The structure of the van der Waals heterostructure is the following from bottom to the top: graphite layer, 54 nm hBN, bilayer graphene, rotated bilayer graphene, 32 nm hBN, aluminum oxide layer, and metallic top gate. Side contacts to the TDBG are created by reactive ion etching and evaporation of 10/50 nm Cr/Au.

### Transport Measurements

Transport measurements were made with AC voltage excitation of 0.1 mV using lock-in technique at 177.13 Hz. The charge density (*n*) and electric displacement field (*D*) were calculated from the gate voltages which is detailed in the Supporting Information.

### Pressurization

Our device was measured using a special high-pressure sample holder equipped with a circuit board suitable for transport measurements of nanodevices at cryogenic temperature, and a piston–cylinder hydrostatic pressure cell. In the cell the pressure was mediated with kerosene, and it was applied using a hydraulic press at room temperature. One of the unique features of our device is that we can use wire bonding to contact the device. Our pressure cell is described in more detail in ref (54). After releasing the pressure, we remeasured some data at ambient pressure which were the same before the pressurization.

### Numerical Calculations

The band structure of the TDBG was calculated using a continuum model. The effect of the pressure is captured by tuning the interlayer couplings modeled in ref (20). The details of our calculations can be found in the Supporting Information.

## Supporting Information Available

Details of device fabrication, optical image of the device, measurement setup, *n*–*D* conversion, determination of lever arms, magnetic oscillations measurements for determining the twist angle, bias measurements, details of band structure calculation, resistance measurements in magnetic field, the gap values for different magnetic field and temperature dependence of the correlated phases, and comparison between the measurements and the model (PDF)

## Notes

Source data of the measurements and the Python code for the simulation are publicly available at https://doi.org/10.5281/zenodo.5532887.
