# Supplementary material for: Tailoring the Band Structure of Twisted Double Bilayer Graphene with Pressure
Source: Nano Lett. 2021 Oct 18;21(20):8777–84. doi: 10.1021/acs.nanolett.1c03066 (PMC8554798; doi:10.1021/acs.nanolett.1c03066)
Supplement: Supplementary file 1 — nl1c03066_si_001.pdf [file nl1c03066_si_001.pdf]

Supporting Information: Tailoring the band structure of twisted double bilayer graphene with pressure

Bálint Szentpéteri,[†] Peter Rickhaus,[‡] Folkert K. de Vries,[‡] Albin Márffy,[†] Bálint Fülöp,[†] Endre Tóvári,[†] Kenji Watanabe,[¶] Takashi Taniguchi,[§] Andor Kormányos,[‖] Szabolcs Csonka,[*,†] and Péter Makk[*,†]

†*Department of Physics, Budapest University of Technology and Economics and Nanoelectronics Momentum Research Group of the Hungarian Academy of Sciences, Budafoki ut 8, 1111 Budapest, Hungary*

‡*Solid State Physics Laboratory, ETH Zürich, CH-8093 Zürich, Switzerland*

¶*Research Center for Functional Materials, National Institute for Materials Science, 1-1 Namiki, Tsukuba 305-0044, Japan*

§*International Center for Materials Nanoarchitectonics, National Institute for Materials Science, 1-1 Namiki, Tsukuba 305-0044, Japan*

‖*Department of Physics of Complex Systems, Eötvös Loránd University, Pázmány P. s. 1/A, 1117 Budapest, Hungary*

E-mail: csonka.szabolcs@ttk.bme.hu; makk.peter@ttk.bme.hu

Device fabrication

The van der Waals heterostructure is fabricated using the dry-transfer technique.[S1] Half of a single BLG is picked up with the top hBN layer ($\sim 32\,\mathrm{nm}$), whereafter the remaining BLG

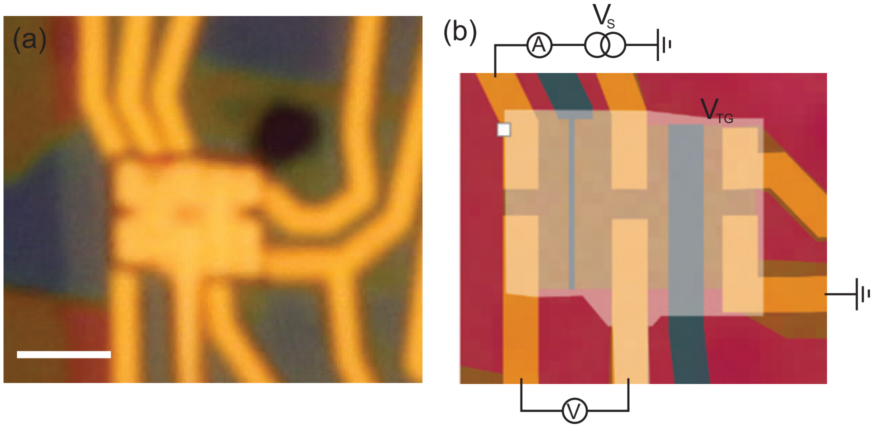

Figure S1: (a) Optical microscope image of the measured device. The scale bar is 2 µm (b) Schematic device geometry showing the four-terminal measurement setup.

is rotated by 1° and subsequently picked up. Then the bottom hBN (∼54 nm) and graphite layer, that serves as a global bottom gate, are added. The device is fabricated in a cleanroom facility, using standard electron beam lithography techniques to define the different layers. Side contacts to the twisted double bilayer graphene (TDBG) are created by reactive ion etching and evaporation of 10 nm of chromium (Cr) and 50 nm of gold (Au). After this the first top gate layer (blue in Fig. S1b) is added, consisting of 10/70 nm of Cr/Au, and the device boundaries are defined by reactive ion etching (red in Fig. S1b). Finally, atomic layer deposition is used to create a 30 nm aluminium oxide dielectric layer, which isolates the second top gate layer of 10/110 nm Cr/Au (grey transparent in Fig. S1b) from the device and first top gate layer. The two top gate layers are used as one in the measurements by adjusting their potential such that the density in the TDBG is constant throughout the device.

Transport measurements

Transport measurements were carried out in a four-terminal and two-terminal geometry with typical AC voltage excitation of 0.1 mV using a standard lock-in technique at 177.13 Hz. The device was cooled down six times: twice at zero pressure, three times at $p = 2$ GPa and once

at $p = 1\,\mathrm{GPa}$. Twice at $p = 2\,\mathrm{GPa}$ we measured in a two-terminal geometry while for the rest of the cooldowns we measured in a four-terminal geometry as depicted in Fig. S1b. In Fig. 3d of the main text the two-terminal measurements are shown with diamond symbols and the four-terminal measurements are shown with rectangular symbols. The measurements in different cooldowns with the same pressure resulted in similar results as it is shown in Fig. S2 for $p = 0$ before applying the pressure and after releasing it.

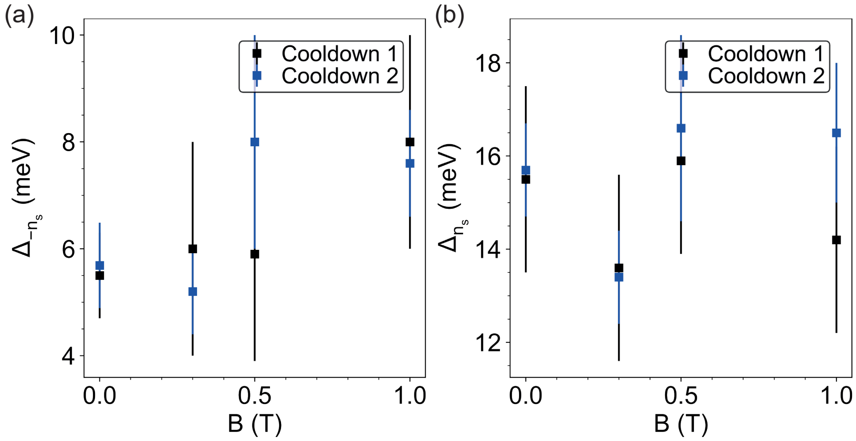

Figure S2: Comparison between the measured gap energies at two different cooldowns at ambient pressure. Cooldown 1 is measured before applying the pressure and cooldown 2 is measured after releasing the pressure.

Gate voltage n-D conversion

The charge density (n) and electric displacement field (D) is related to the top and bottom gate voltage by

$$\begin{aligned} n &= \alpha_{\mathrm{TG}} V_{\mathrm{TG}} + \alpha_{\mathrm{BG}} V_{\mathrm{BG}} + n_0 \\ \frac{D}{\epsilon_0} &= \frac{e}{2\epsilon_0} \left(\alpha_{\mathrm{TG}} V_{\mathrm{TG}} - \alpha_{\mathrm{BG}} V_{\mathrm{BG}}\right) + \frac{D_0}{\epsilon_0}, \end{aligned} \tag{S1}$$

where α_{TG} and α_{BG} are the lever arm for the top and bottom gate respectively, ϵ_0 is the vacuum permittivity, V_{TG} and V_{BG} are the top and bottom gate voltage, respectively, $n_0 = -\alpha_{\mathrm{TG}} V_{\mathrm{TG0}} - \alpha_{\mathrm{BG}} V_{\mathrm{BG0}}$ is the carrier density when the gate voltages are set to zero,

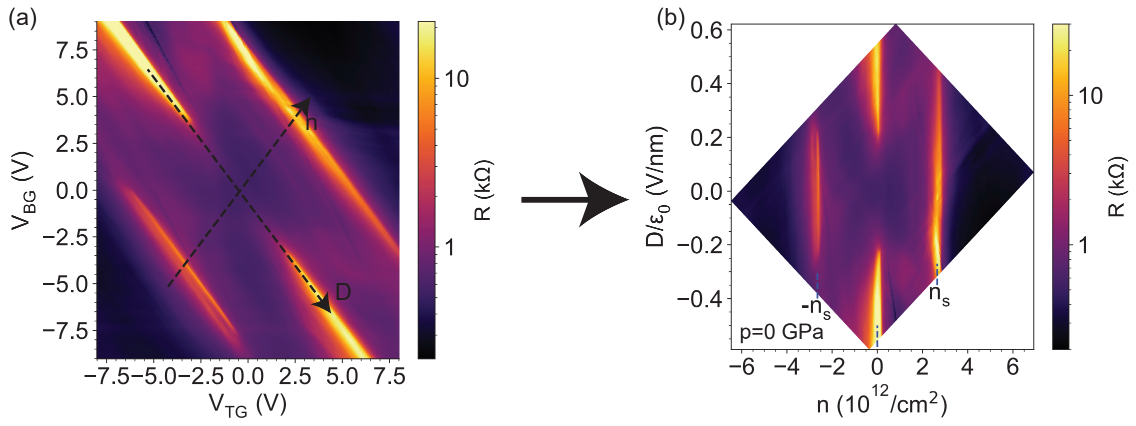

Figure S3: Bottom and top gate voltage conversion to n and D.

whereas $D_0 = -\frac{e}{2}(\alpha_{\text{TG}} V_{\text{TG0}} - \alpha_{\text{BG}} V_{\text{BG0}})$ is a built-in offset electric field. V_{TG0} and V_{BG0} are the values of the top and bottom gate at the zero density and zero displacement field point.

Table S1: The extracted lever arms at different pressures.

P (GPa)	0	1	2
α_{TG} $(\frac{10^{15}}{\text{Vm}^2})$	3.38(5)	3.75(5)	3.84(5)
α_{BG} $(\frac{10^{15}}{\text{Vm}^2})$	4.54(6)	4.94(7)	4.91(8)
n_0 (10^{15}/m)	2.3(2)	2.5(2)	2.4(2)
D_0 (V/nm)	0.016(5)	0.018(5)	0.017(5)

The lever arms were obtained from the gate-gate maps (Fig. S3a) and from the quantum oscillations in magnetoconductance measurements (Fig. S4). The extracted lever arms are shown in Table S1. The pressure dependence of the lever arms is originating from the compression of the dielectrics and pressure-dependent dielectric constant of the hBN as already reported in Refs. S2,S3. The extracted lever arms within the margin of error were the same at the same pressures at different cooldowns.

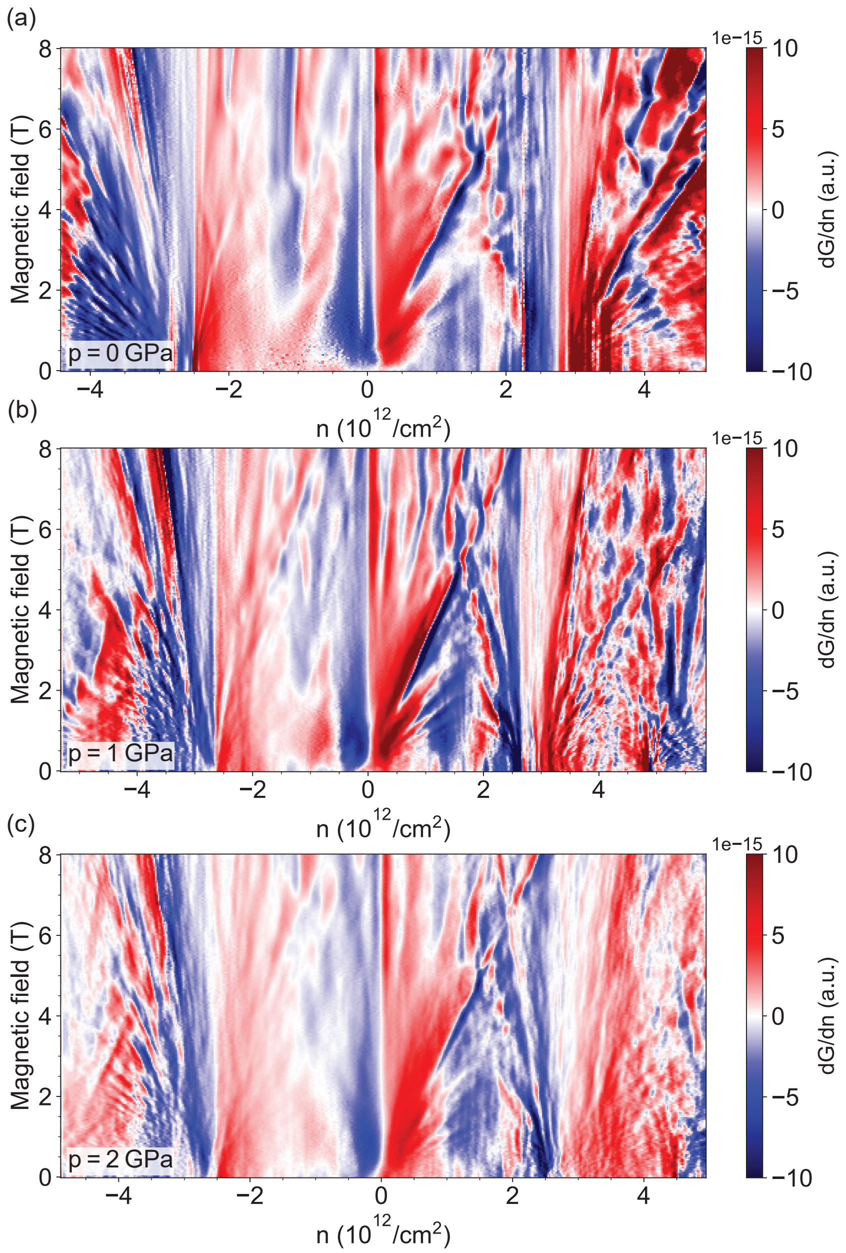

Figure S4: Magnetic oscillation measurements of the four-terminal conductance G. (a), (b), (c) show $\mathrm{d}G/\mathrm{d}n$ versus n and B at $p = 0$, $1\,\mathrm{GPa}$ and $2\,\mathrm{GPa}$ respectively.

Pressure dependence of the twist angle

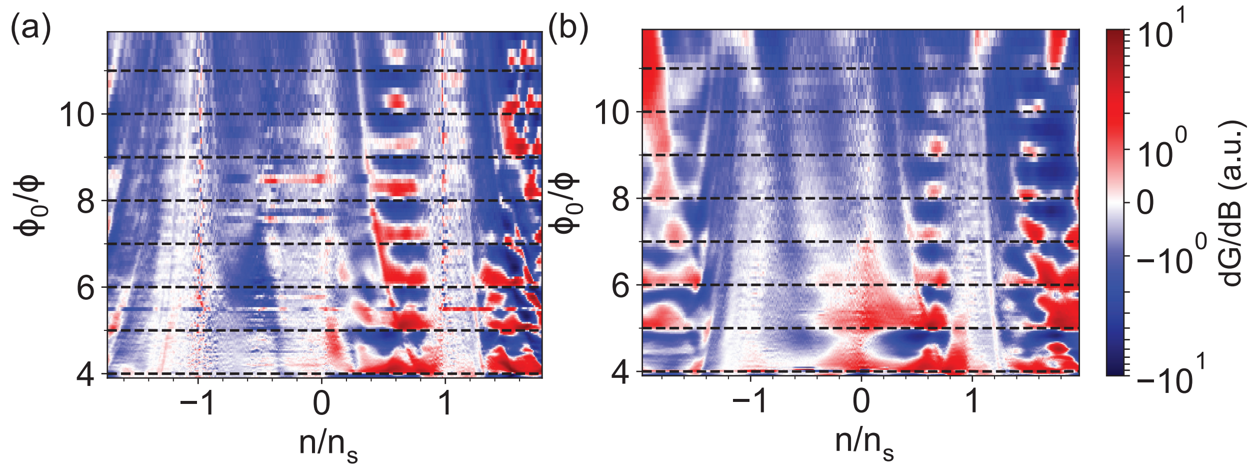

Figure S5: Brown-Zak oscillations. (a) 2D color map of $\mathrm{d}G/\mathrm{d}B$ versus n/n_s and Φ_0/Φ at $D = 0$ and at ambient pressure. (b) 2D color map of $\mathrm{d}G/\mathrm{d}B$ versus n/n_s and Φ_0/Φ at $D = 0$ under 2 GPa pressure. On the figures, the oscillating pattern is well observable and their periodicity is the same. The dashed lines are a guide to the eye for the oscillations.

At certain magnetic fields, the magnetic length is commensurable with the lattice periodicity which results in the recovery of the translation symmetry thus the electron feels effectively zero magnetic field. This results an oscillation in the resistance called Brown-Zak (BZ) oscillation.[S4,S5] A common method to determine the twist angle (ϑ) in superlattice structures such as the TDBG via transport measurements is to calculate it from the BZ oscillations. It can be calculated using that the BZ oscillations have maxima in the conductance at $\phi = BA_\mathrm{s} = \frac{\phi_0}{q}$, where $\phi_0 = h/e$ is the flux quantum, q is an integer number and A_s is the area of the superlattice unit cell which is given by

$$A_\mathrm{s} = \frac{\sqrt{3}}{2}\left(\frac{a}{2\sin(\vartheta/2)}\right)^2, \tag{S2}$$

where a is the lattice constant of the graphene. We determined the twist angle ($\vartheta = 1.067°\pm0.003°$) from our data at different pressures and found that they were identical within the uncertainty of our measurements.

We also estimated the twist angle inhomogeneity from the width of the resistance peaks at $n = \pm n_s$ in Fig. S7 panel a d and f at different pressures which varied in a similar range: at $p = 0$ GPa ϑ is between 1.06° and 1.09°, at $p = 1$ GPa ϑ is between 1.06° and 1.1° and at $p = 2$ GPa ϑ is between 1.05° and 1.08°. In regions containing substantial twist angle inhomogeneity, this inhomogeneity could result in smaller measured gaps values, but it doesn't change our results qualitatively.

Bias measurements

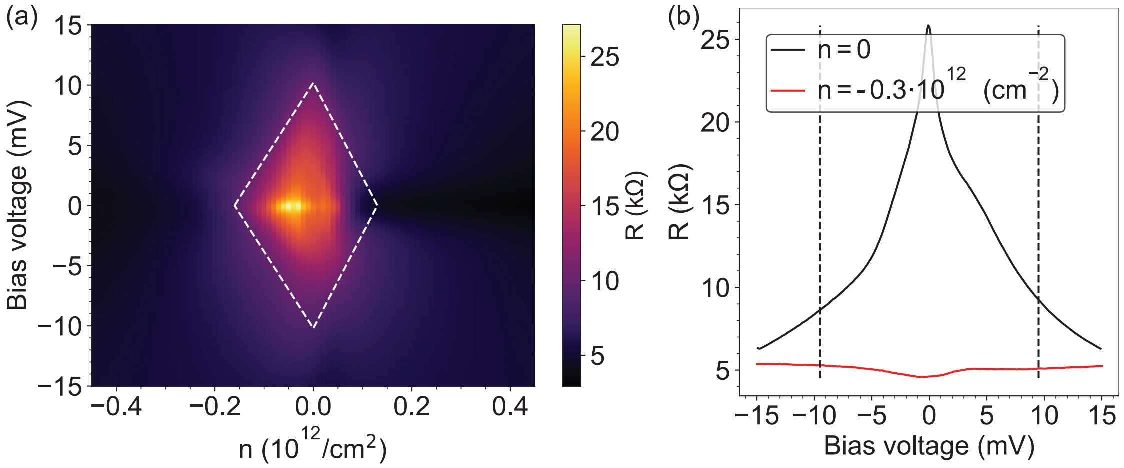

Figure S6: Bias measurements. (a) The resistance versus n and the bias voltage (V_B) at $D = -0.3$ V/nm. (b) Line-cut from (a) at $n = 0$ and $n = -3 \cdot 10^{11}$ 1/cm^2. The gaps were obtained by taking the bias voltage where the resistance drops to 15% of the highest value at $V_B = 0$, after subtracting a background.

At the CNP we also estimated the gaps with bias measurements. We measured the two-terminal resistance of the device with the lock-in technique at low frequency and applied a DC voltage bias (V_B) to the sample. If we apply a higher or equal bias voltage to the gap, transport processes become available. We performed bias measurements over a small range of n at fix D (see Fig. S6a). First the maximum resistance (R_{max}) was determined (occurring in Fig. S6a at $n = 0$ and $V_B = 0$). We defined the gap value as the point where the resistance drops to 15% of the maximum resistance value, also taking into account a

background value. To determine the gap size, the following procedure was followed. At the gap ($n = 0$, $V_\mathrm{B} = 0$) we took the peak value (R_max), while at a slightly different density value, where the resistance R is virtually independent of V_B, we took the background value R_gapless. We approximated the gap energy with $e|V_\mathrm{B}|$ where $V_\mathrm{B} = (V_\mathrm{B+} + V_\mathrm{B-})/2$ with $R(\pm V_\mathrm{B\pm}) = R_\mathrm{gapless} + 0.15(R_\mathrm{max} - R_\mathrm{gapless})$ as it is shown in Fig. S6b.

Band structure calculation

The band structure of TDBG is calculated using the non-interacting Bistritzer-Macdonald model [S6] and applied to the TDBG using the parameters from Ref. S7. The matrix elements in the model can be written as

$$\begin{aligned}\langle ls\mathbf{k}|H|l's'\mathbf{k}'\rangle &= \delta_{l,l'}H_{ls,l's'}(\mathbf{k})\delta_{\mathbf{k},\mathbf{k}'} + (1-\delta_{l,l'})H_{ls,l's'}(\mathbf{K})\left[\delta_{\mathbf{k},\mathbf{k}'} + \right.\\ &\left. e^{-i(\xi\mathbf{b}_1\tau_s - \xi\mathbf{b}'_1\tau_{s'})}\delta_{\mathbf{k}-\xi\mathbf{b}_1,\mathbf{k}'-\xi\mathbf{b}'_1} + e^{-i(\xi\mathbf{b}_2\tau_s - \xi\mathbf{b}'_2\tau_{s'})}\delta_{\mathbf{k}-\xi\mathbf{b}_2,\mathbf{k}'-\xi\mathbf{b}'_2}\right],\end{aligned} \tag{S3}$$

where $\delta_{l,l'}$ is the Kronecker delta fuction, $l = \{\mathrm{top}, \mathrm{bottom}\}$ is the layer index of the BLGs, $s = \{A, B, A', B'\}$ is the site index within the BLG, $\mathbf{k}$ is a wave-vector in the $\mathbf{k}$-space, $H_{ls,l's'}(\mathbf{k})$ is the low-energy Hamiltonian of the BLG, $\mathbf{K}$ is the Dirac-point wave vector, $\xi = \pm 1$ for $\mathbf{K}$ and $\mathbf{K}'$, $\mathbf{b}_i$ and $\mathbf{b}'_i$ are the primitive reciprocal lattice vectors for the top and bottom BLGs and τ_s are the sublattice positions.

In our calculations we used a configuration space with cutoff in momentum space with a radius up to $4|b_\mathrm{s}| = 32\sin(\vartheta/2)/(\sqrt{3}a)$ using Hamiltonian matrices with a size of 648×648. In the model for the low-energy Hamiltonian of the BLG ($\delta_{l,l'}H_{ls,l's'}(\mathbf{k})$) we included the remote hoppings and used the following parameters: $\gamma_0 = 3.1$ eV for the intralayer nearest-neighbor hopping, $\gamma_1 = 3\omega(p)$ eV for the interlayer coupling between orbitals on the dimer sites, $\gamma_3 = 0.283$ eV for the interlayer coupling between orbitals on the non-dimer sites, $\gamma_4 = 0.138$ eV for the interlayer coupling between dimer and non-dimer orbitals and $\delta = 0.015$ eV onsite energy difference between dimer and non-dimer sites. [S7,S8] For the matrix elements between

the BLGs ($[1-\delta_{l,l'}]H_{ls,l's'}(\mathbf{K})$) which are given by

$$H_{ls,l's'}(\mathbf{K}) = \begin{pmatrix} \omega'(p) & \omega(p) \\ \omega(p) & \omega'(p) \end{pmatrix}, \tag{S4}$$

the pressure dependent interlayer couplings $\omega(p)$ and $\omega'(p)$ were taken from Table I. in Ref. S7 as

$$\omega(p) = A + \sqrt{B + C \cdot p}, \tag{S5}$$

where $A = 0.0546$ $B = 0.0044$ $C = 0.0031$ for ω and $A = 0.0561$ $B = 0.0018$ $C = 0.0018$ for ω'.

The external electric field modifies the layer potentials (u_i), where $i = \{1, 2, 3, 4\}$ marks the 4 layers of graphene from top to bottom. The effect of the external electric field is modelled with $u_1 = -u_4$, $u_2 = -u_3$, and we introduce u as the potential difference within each BLG and $2u'$ as the potential difference between the BLGs as $u_1 = (u + u')/2$ and $u_2 = (-u + u')/2$. In the calculations, we modeled the external electric field with equal interlayer potential drops ($u' = 2u$) and neglected the quantum capacitance corrections and the electron-electron interactions, which could modify the low-energy flat bands.

Magnetic field dependence

In Fig. S7 we present n-D resistance maps at $B_\mathrm{z} = 0\,\mathrm{T}$, $1\,\mathrm{T}$ and $2\,\mathrm{T}$ at $1.5\,\mathrm{K}$. At ambient pressure a gap opens at the charge density of $n = n_\mathrm{s}/2$ which corresponds to a correlated insulating phase.[S9–S11] In panel d,e and f,g the measurements are shown for 0 and $2\,\mathrm{T}$ at $1\,\mathrm{GPa}$ and $2\,\mathrm{GPa}$, respectively. Under pressure this gap disappears for all applied B_z fields.

In Fig. S8 we show resistance maps at various in-plane magnetic fields (B_x) at $p = 2\,\mathrm{GPa}$. Comparing $B_\mathrm{x} = 0$ (panel a) and $B_\mathrm{x} = 3\,\mathrm{T}$ (panel b) it is visible that the effect of the in-plane magnetic field is negligible. At a finite perpendicular magnetic field, the effect of applying an in-plane field is also negligible. In thermal activation measurements, B_x also had a negligible

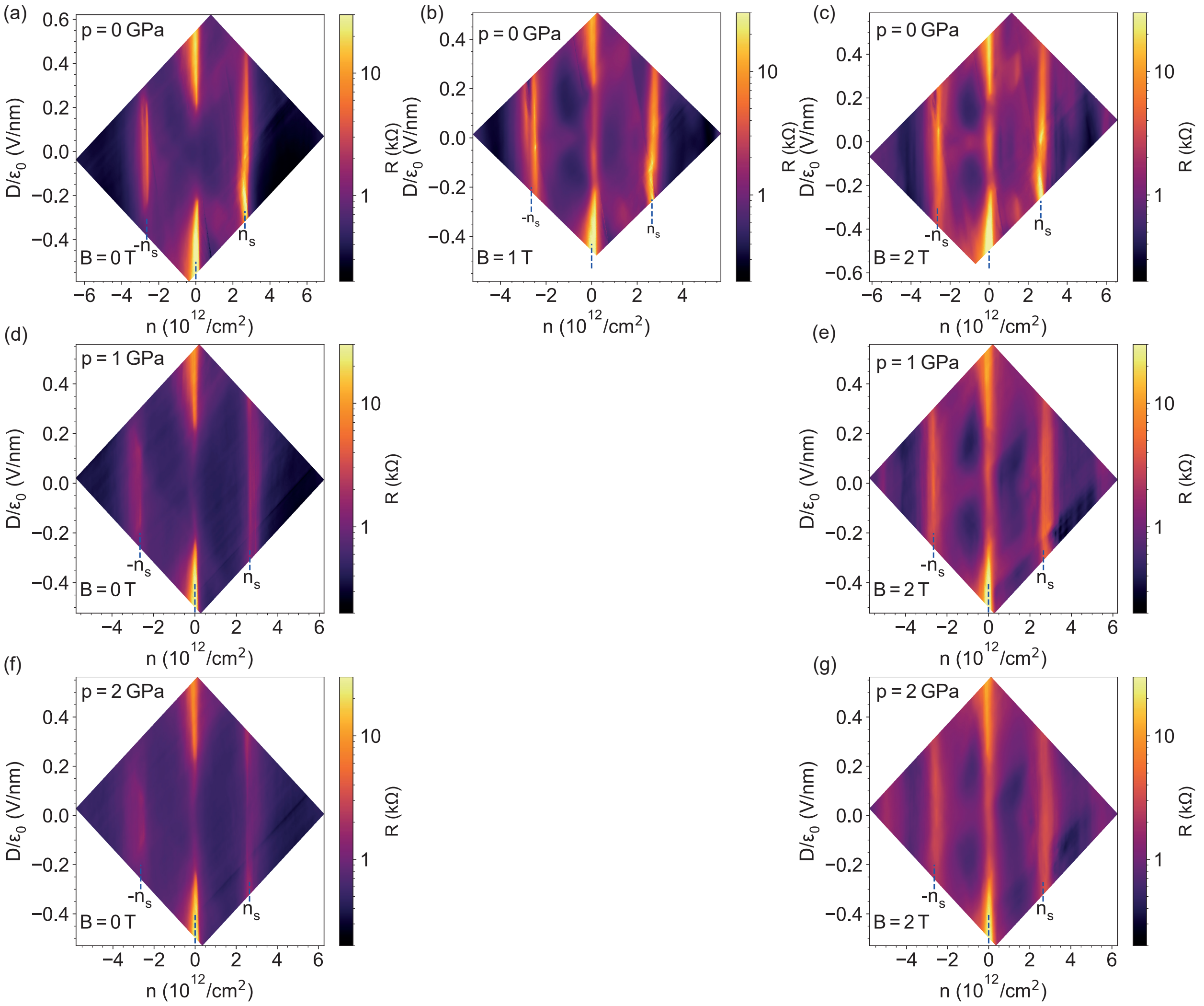

Figure S7: Four-probe resistance of the TDBG as a function of the charge density (n) and electric displacement field (D) measured in (a-c) at ambient pressure, in (d) and (e) at $p = 1\,\mathrm{GPa}$ and in (f) and (g) at $p = 2\,\mathrm{GPa}$ at different magnetic fields.

effect on the extracted gaps.

In Fig. S9 panel a, b the vertical magnetic field dependence of the measured moiré band gaps is shown. $\Delta_{-\mathrm{n_s}}$ increases with B at $D = 0$ for both $p = 0$ and $p = 2\,\mathrm{GPa}$ (shown in panel a). The same tendency is visible for $\Delta_{\mathrm{n_s}}$ at $2\,\mathrm{GPa}$ and at $D = 0$, but at $p = 0$ $\Delta_{\mathrm{n_s}}$ seems independent of B in a limited magnetic field range as depicted in panel b. Δ_{CNP} at finite D is independent of B at small magnetic fields as it is shown in Fig. S9c. Fig. S9d shows the magnetic field dependence of the gaps at the CNP for different pressures for $D = 0$. At

$p = 0$ only measurements up to 4 T were available. These findings are similar to Ref. S12 and are discussed in the main text. We note that our model does not include the effect of magnetic fields and more advanced model capable of handling that goes beyond the scope of this manuscript.

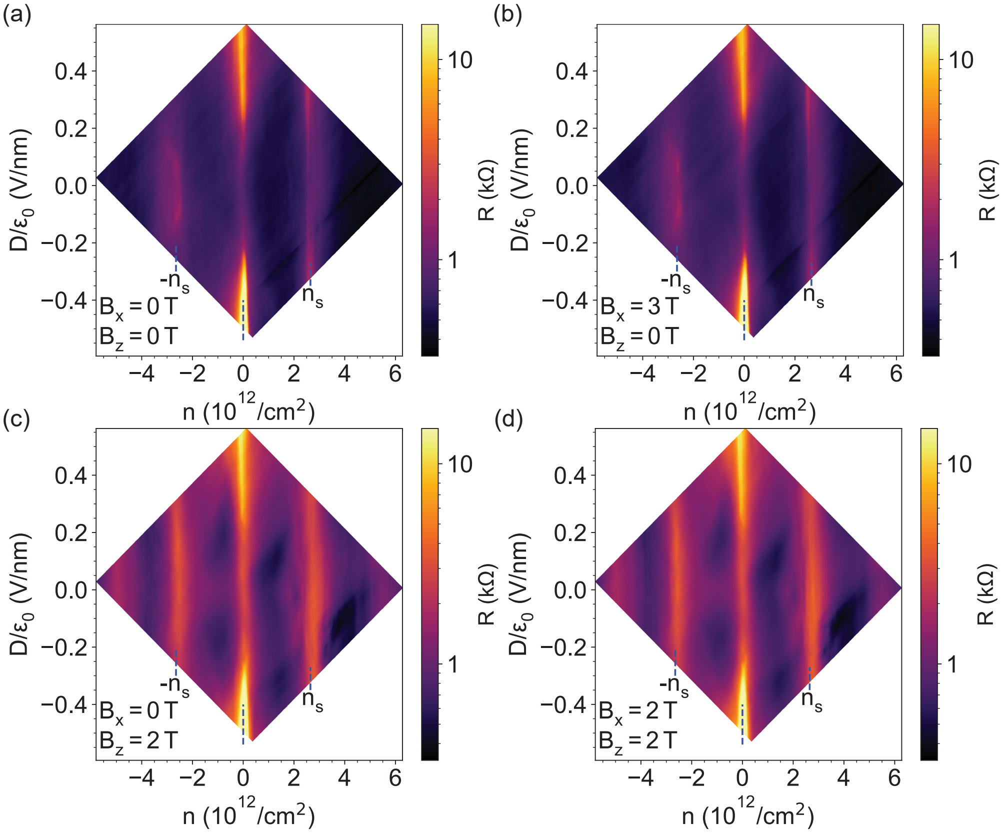

Figure S8: Four-probe resistance of the TDBG as a function of n and D measured at 2 GPa in different in- and out-plane magnetic fields. (a) presents the data for zero magnetic field, (b) for $B_{\mathrm{x}} = 3\,\mathrm{T}$ in-plane magnetic field, (c) for $B_{\mathrm{z}} = 2\,\mathrm{T}$ vertical magnetic field whereas (d) shows the measurement in $B_{\mathrm{z}} = 2\,\mathrm{T}$ and $B_{\mathrm{x}} = 2\,\mathrm{T}$.

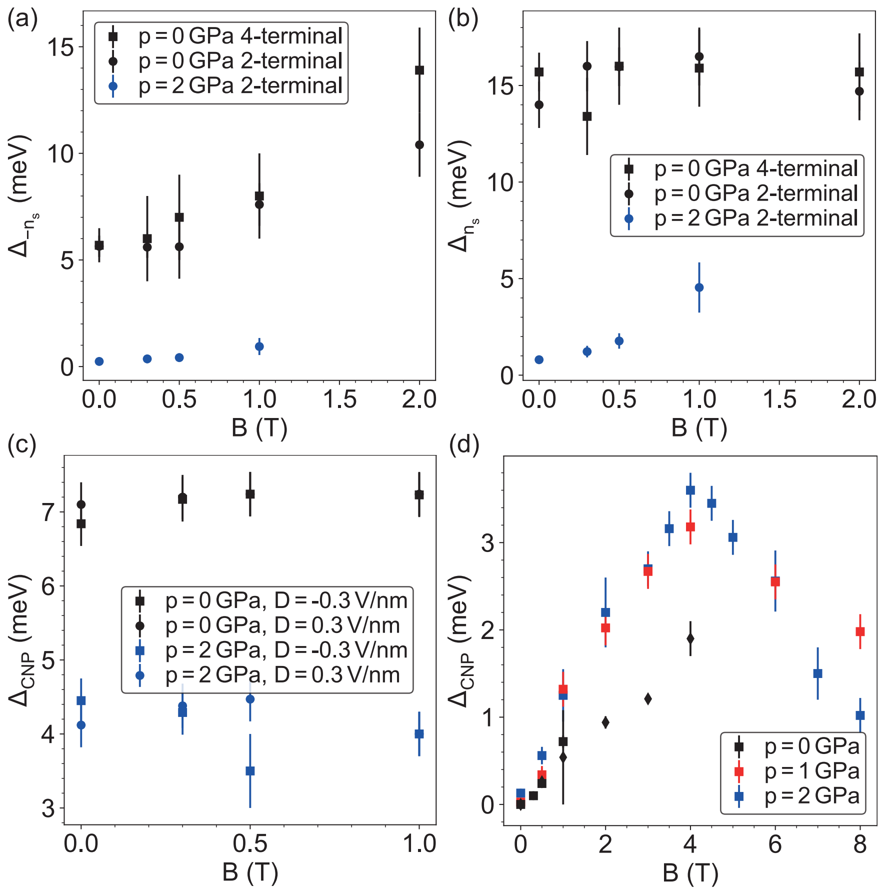

Figure S9: Out-of-plane magnetic field dependence of the band gaps. (a) $\Delta_{-\mathrm{n_s}}$ versus B at $D = 0$ at $p = 0$ and at $p = 2\,\mathrm{GPa}$. (b) $\Delta_{\mathrm{n_s}}$ versus B at $D = 0$ at $p = 0$ and at $p = 2\,\mathrm{GPa}$. (c) Δ_{CNP} at D/ϵ_0=0.3 V/nm and -0.3 V/nm shown with squares and circles respectively. (d) Δ_{CNP} at $D = 0$.

Temperature dependence at half and three-quarter fillings

Fig. S10 show the temperature dependence of the correlated states at half and near three-quarter fillings which are similar what was observed in Ref. S10,S11,S13,S14. At finite pressure the correlated states disappear as shown in Fig. S7.

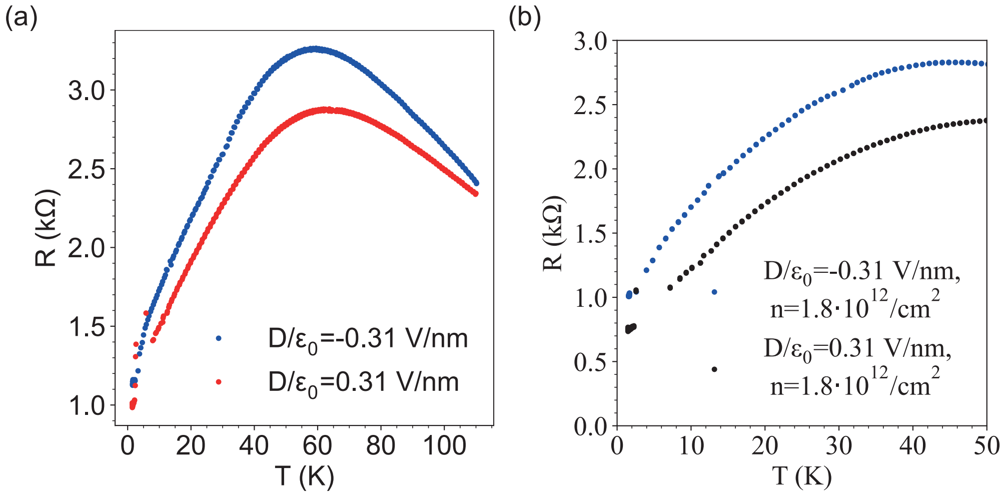

Figure S10: Temperature dependence of the resistance at the correlated phases. (a) $R(T)$ at $n = \mathrm{n_s}/2$ at two different displacement fields. (b) $R(T)$ near three-quarter filling.

Comparison between the measurement and the model

To compare experimental findings with our calculations we used the relation of $\frac{D}{\epsilon_0} = eu\frac{\epsilon}{d}$, where $d = 0.33\,\mathrm{nm}$ is the interlayer distance of bilayer graphene and ϵ is the relative dielectric constant of bilayer graphene. We also used the same conversion between the top layer of the bottom BLG and the bottom layer of the twisted, top BLG. A qualitatively good agreement at the CNP is achieved using $\epsilon = 5$ which is depicted in Fig. S11 where the experiments and the theory are shown in the same figure.

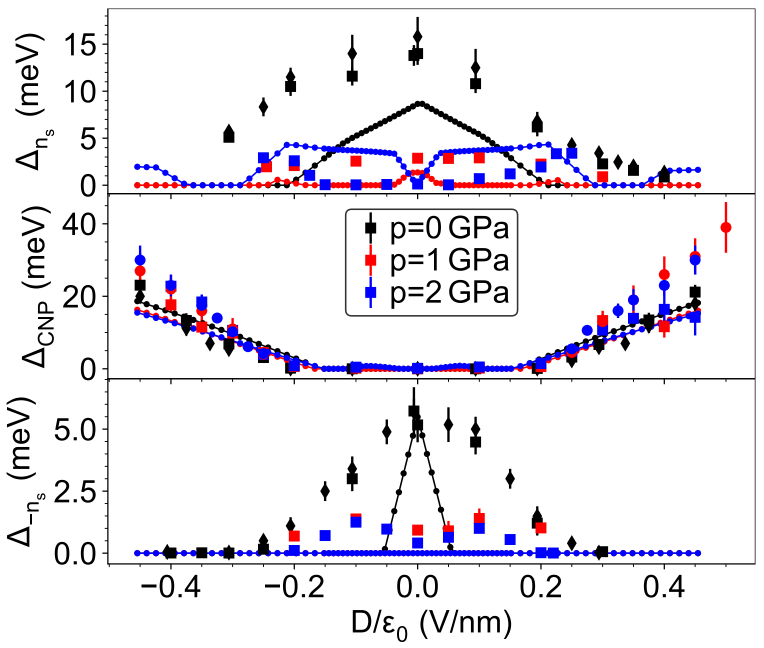

Figure S11: Comparison between the measurement and the model. The displacement field was calculated from the interlayer potential with $\frac{D}{\epsilon_0} = eu\frac{\epsilon}{d}$ with $\epsilon = 5$.

References

[S1] Kim, K.; Yankowitz, M.; Fallahazad, B.; Kang, S.; Movva, H. C. P.; Huang, S.; Larentis, S.; Corbet, C. M.; Taniguchi, T.; Watanabe, K.; Banerjee, S. K.; LeRoy, B. J.; Tutuc, E. van der Waals Heterostructures with High Accuracy Rotational Alignment. *Nano Letters* **2016**, *16*, 1989–1995.

[S2] Solozhenko, V.; Will, G.; Elf, F. Isothermal compression of hexagonal graphite-like boron nitride up to 12 GPa. *Solid State Communications* **1995**, *96*, 1–3.

[S3] Yankowitz, M.; Jung, J.; Laksono, E.; Leconte, N.; Chittari, B. L.; Watanabe, K.; Taniguchi, T.; Adam, S.; Graf, D.; Dean, C. R. Dynamic band-structure tuning of graphene moiré superlattices with pressure. *Nature* **2018**, *557*, 404–408.

[S4] Brown, E. Bloch Electrons in a Uniform Magnetic Field. *Physical Review* **1964**, *133*, A1038–A1044.

[S5] Zak, J. Magnetic Translation Group. *Physical Review* **1964**, *134*, A1602–A1606.

[S6] Bistritzer, R.; MacDonald, A. H. Moire bands in twisted double-layer graphene. *Proceedings of the National Academy of Sciences* **2011**, *108*, 12233–12237.

[S7] Chebrolu, N. R.; Chittari, B. L.; Jung, J. Flat bands in twisted double bilayer graphene. *Physical Review B* **2019**, *99*, 235417.

[S8] Jung, J.; MacDonald, A. H. Accurate tight-binding models for theπbands of bilayer graphene. *Physical Review B* **2014**, *89*, 035405.

[S9] Burg, G. W.; Zhu, J.; Taniguchi, T.; Watanabe, K.; MacDonald, A. H.; Tutuc, E. Correlated Insulating States in Twisted Double Bilayer Graphene. *Physical Review Letters* **2019**, *123*, 197702.

[S10] Shen, C. et al. Correlated states in twisted double bilayer graphene. *Nature Physics* **2020**, *16*, 520–525.

[S11] Liu, X.; Hao, Z.; Khalaf, E.; Lee, J. Y.; Ronen, Y.; Yoo, H.; Najafabadi, D. H.; Watanabe, K.; Taniguchi, T.; Vishwanath, A.; Kim, P. Tunable spin-polarized correlated states in twisted double bilayer graphene. *Nature* **2020**, *583*, 221–225.

[S12] Burg, G. W.; Lian, B.; Taniguchi, T.; Watanabe, K.; Bernevig, B. A.; Tutuc, E. Evidence of Emergent Symmetry and Valley Chern Number in Twisted Double-Bilayer Graphene. 2020; 2006.14000. arXiv. `https://arxiv.org/abs/2006.14000` (date accessed: 09/10/2021).

[S13] Cao, Y.; Rodan-Legrain, D.; Rubies-Bigorda, O.; Park, J. M.; Watanabe, K.; Taniguchi, T.; Jarillo-Herrero, P. Tunable correlated states and spin-polarized phases in twisted bilayer–bilayer graphene. *Nature* **2020**, *583*, 215–220.

[S14] He, M.; Li, Y.; Cai, J.; Liu, Y.; Watanabe, K.; Taniguchi, T.; Xu, X.; Yankowitz, M.

Symmetry breaking in twisted double bilayer graphene. *Nature Physics* **2020**, *17*, 26–30.
